# The Effect of Hyperbaric Oxygen Therapy on Functional Impairments Caused by Ischemic Stroke

**DOI:** 10.1155/2018/3172679

**Published:** 2018-10-09

**Authors:** Emily R. Rosario, Stephanie E. Kaplan, Sepehr Khonsari, Garrett Vazquez, Niyant Solanki, Melanie Lane, Hiriam Brownell, Sheila S. Rosenberg

**Affiliations:** Casa Colina Hospital and Centers for Healthcare, Pomona CA, 255 East Bonita Avenue, Pomona, CA 91767, USA

## Abstract

**Background:**

While research suggests a benefit of hyperbaric oxygen therapy (HBOT) for neurologic injury, controlled clinical trials have not been able to clearly define the benefits.

**Objective:**

To investigate the effects of HBOT on physical and cognitive impairments resulting from an ischemic stroke.

**Methods:**

Using a within-subject design a baseline for current functional abilities was established over a 3-month period for all subjects (n=7). Each subject then received two 4-week periods of HBOT for a total of 40 90-minute treatments over a 12-week period. Subjects completed a battery of assessments and had blood drawn six times over the 9-month total duration of the study.

**Results:**

We found improvements in cognition and executive function as well as physical abilities, specifically, improved gait. Participants reported improved sleep and quality of life following HBOT treatment. We also saw changes in serum levels of biomarkers for inflammation and neural recovery. In the functional domains where improvement was observed following HBOT treatment, the improvements were maintained up to 3 months following the last treatment. However, the physiological biomarkers showed a pattern of more transient changes following HBOT treatment.

**Conclusions:**

Findings from this study support the idea of HBOT as a potential intervention following stroke.

## 1. Introduction

Each year over 795,000 Americans will suffer a stroke resulting in death or significant disability. While considerable functional gains are often made, significant assistance in daily life is still required in approximately one-third of stroke survivors [1]. Following an ischemic stroke, in which cerebral blood flow is impaired, irreversible neural injury occurs within minutes (for review see [2, 3]). Of particular therapeutic interest are the regions surrounding the focal site of injury where the tissue is at risk but not facing irreparable damage, and the potential to salvage these neurons still exists [4–6]. Imaging has shown that those at risk regions may persist in a dysfunctional state for months to years after the injury [7, 8]. Cell death and reduced neuronal activity resulting from an ischemic event can be attributed to excitotoxicity, oxidative stress, inflammation, and apoptosis, which are all pathways where hypoxia plays a key role (For review see [5]). Decreased oxygenation to the damaged area including blood vessels further prevents tissue repair and the generation of new synaptic connections [8, 9]. Consequently, increased oxygen has been considered as a potential treatment for stroke for several decades [10].

Hyperbaric oxygen can be defined as the breathing of 100% oxygen at a pressure higher than atmospheric pressure. Initially, hyperbaric oxygen therapy (HBOT) was used to treat decompression sickness in divers; however, over the years its far-reaching potential was recognized, and it has been approved for a variety of purposes including wound repair, carbon monoxide poisoning, anemia, thermal burns, delayed radiation injuries, osteomyelitis, and actinomycosis (for review see [11]). In addition to these conditions, there has been a great deal of interest in the use of HBOT for brain injury, stroke, and cerebral palsy. The use of HBOT for brain injury is based on the hypothesis that injured or inactive neurons would benefit from increased blood flow and oxygen delivery, which would act to metabolically or electrically reactivate the cells [8, 12–15]. However, while HBOT is approved for several clinical indications [16], the effects of HBOT in the brain have yet to be clearly defined.

Supporting a role for the use of HBOT in stroke patients is a wealth of experimental studies in a number of different animal models [17–22]. Specifically, in a variety of animal models including young and aged rats, mice, rabbits, and dogs a number of different experimental paradigms have been used to induce stroke-like neural injury. In these different models, HBOT has been shown to decrease intracranial pressure, reduce blood-brain-barrier permeability and cerebral edema [23], increase cellular metabolism, stabilize levels of glutamate, glucose, and pyruvate, attenuate inflammatory response [24], and increase antiapoptotic* bcl* family genes [6, 17, 20, 25–28]. HBOT has also been shown to be efficacious in improving learning and memory deficits in rats [29–31], an effect we predict may be correlated with the cellular and molecular effects contributing to neuronal viability and plasticity [15]. Although observed effects of HBOT vary depending on the experimental conditions (e.g., animal model, type of injury, timing of treatment, duration of treatment HBOT, etc.), animal studies suggest numerous benefits and an underlying mechanism, which warrants continued research in both animals and humans to fully elucidate the benefits of HBOT.

Despite the seemingly overwhelming potential of HBOT as defined by basic research and the underlying mechanistic rationale, clinical investigations have largely not produced the expected results. While research has provided some favorable evidence for HBOT in both acute strokes and poststroke [21, 22, 32–39, 39, 40], methodological issues have limited the interpretation and generalizability of the results [2, 6]. The timing of HBOT after stroke and duration of treatment remain critical questions to be answered. In this preliminary study we attempted to address some of these issues by investigating the use of HBOT as a therapeutic intervention for stroke patients in the chronic stage of their illness using a within subjects design. While the sample was small and not blinded to treatment we predicted that HBOT would result in numerous benefits on a variety of functional impairments that may occur following an ischemic stroke. We assessed a large number of dependent variables to attempt to capture all possible changes within the range of poststroke impairments. Of course, some participants did not show deficits in certain domains prior to treatment; therefore we would not expect a change in those areas.

## 2. Methods

### 2.1. Participant

Seven subjects (4 females) were enrolled in this study; 6 completed the study. Subjects eligible for this study were male or female and any age between 18 and 80 years who had suffered an ischemic stroke at least 12-month ago (to minimize the chance for spontaneous recovery) and exhibited some functional impairments. Of the participants 50% were 1 year after stroke when they enrolled in the study and the other 50% were 2 years after stroke (Table 1). A stable baseline (i.e., no clinically meaningful functional improvement over a 3-month period on measures of cognitive or motor behavior) was established for each participant before he or she could move forward in the study. Significant improvement was noted in some quality of life domains over the 3-month baseline period however, these subjective patient report domains were not enough to prevent moving forward with the study when there were no cognitive or physical functional changes. Subjects were excluded from the study if they satisfied any of the following conditions: hydrocephalus, recurrent stroke, neurologic condition that affect motor or cognitive ability (i.e., Alzheimer's disease, Parkinson's disease, ALS, multiple sclerosis), history of seizures, were receiving thrombolytics, COPD with CO2 retention, pneumothorax, bowel obstruction, sickle cell disease, cardiac arrhythmia, claustrophobia, active alcohol or drug abuse, current participation in physical, occupational, or speech therapy, or extremely severe cognitive impairment.

### 2.2. Study Design

**Figure 1:**
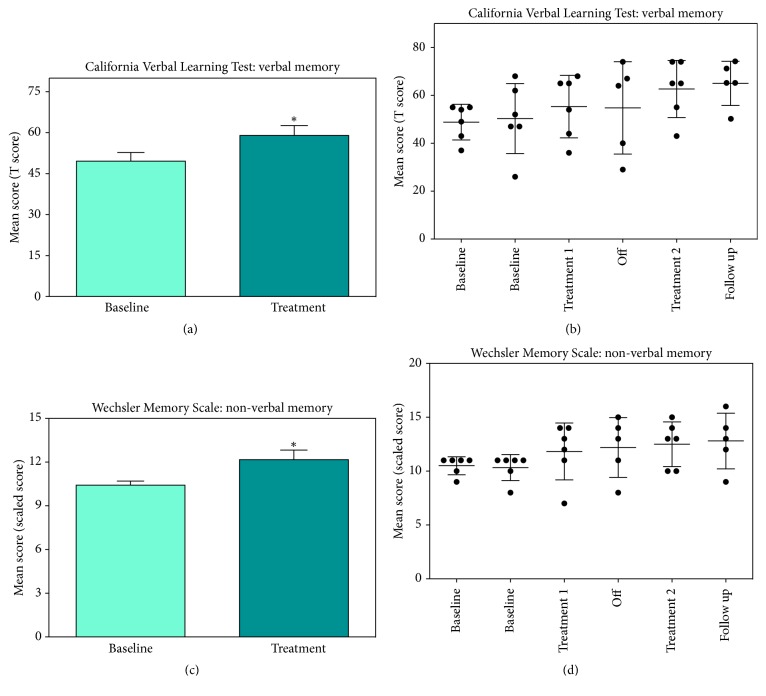
Effect of HBOT on memory: we observed a significant effect of HBOT on verbal and nonverbal memory using the CVLT (a & b), which measures verbal memory, and the WMS (c & d), which measures nonverbal memory. Graphs (a) and (c) represent the difference between baseline and treatment and (b) and (d) show all individual data points.

**Figure 2:**
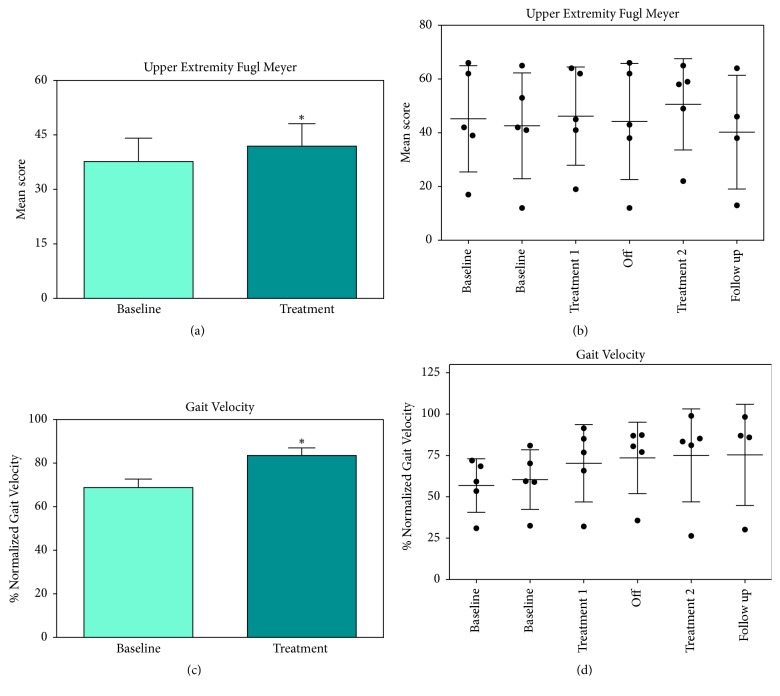
Effect of HBOT on Gait and UE mobility: we observed significant improvement in physical abilities as measured with the Upper Extremity Fugl Meyer (UEFM, (a & b) and gait velocity (c & d). Graphs (a) and (c) represent the difference between baseline and treatment and (b) and (d) show all individual data points.

**Figure 3:**
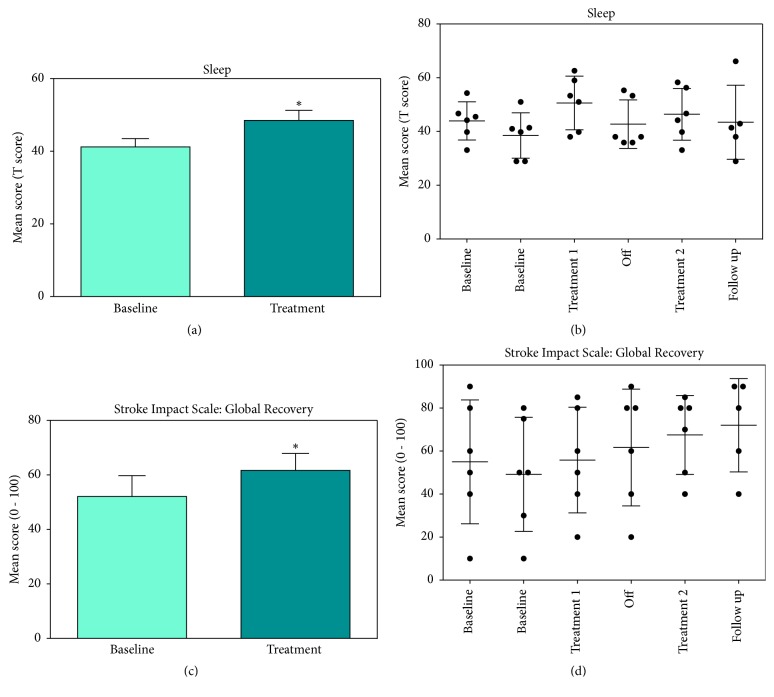
Effect of HBOT on Quality of Life: participants reported significant improvement in sleep (a & b) and overall global recovery (c & d) following HBOT. Graphs (a) and (c) represent the difference between baseline and treatment and (b) and (d) show all individual data points.

To carry out this study we used a within-subject design in which each subject provided his or her own baseline (i.e., pretreatment) comparison. A stable baseline was confirmed by the absence of functional recovery over the previous 3-months. Each subject was tested on a battery of outcome measures (cognitive, physical, speech, and quality of life measures) twice during a 3-month period to determine the baseline and 4 more times throughout the 9-month study to assess the effect of HBOT. If a steady baseline was established (based on the variables measured), the subject was eligible to begin the first round of HBOT treatment. HBOT consisted of 20 treatments of 100% O2 at 2.0 ATA for 60 minutes each day Monday through Friday for a total of 4 weeks. After this first treatment period ended and following four weeks without HBOT (labeled as Off below and in Figures 1–3), data were collected for the same outcome battery. The second round of HBOT treatment was identical to the first in all conditions. Following the second 4 weeks of HBOT outcome, data were collected again. Finally, follow-up testing on all experimental endpoints was completed for each subject 3-months after completion of the second round of HBOT. A physician monitored each subject throughout the 9-month study duration to assess any health issues and potential complications.

### 2.3. Experimental Endpoints

The experimental endpoints for this study included speech measures, neuropsychological measures, physical measures, quality of life measures, and physiological biomarkers.

#### 2.3.1. Language Measures

To assess changes in aphasia, communication ability, language, and verbal fluency the Boston Naming Test (BNT) and Reading Comprehension Battery for Aphasia (RCBA) with latencies were used. The Porch Index of Communication Ability (PICA) scoring was used for all tests, which provides a multidimensional scoring system for communication ability that describes accuracy, responsiveness, completeness, promptness, and efficiency of each response.

#### 2.3.2. Neuropsychological Measures

Cognitive and behavioral impairments (e.g., memory, attention/concentration, verbal fluency, and depression) were assessed using the Mini-Mental Status Exam (MMSE), California Verbal Learning test (CVLT-II), Grooved Pegboard test (GP), Trails A and B, Controlled Oral Word Association test (COWAT), Semantic Fluency (SF, animals), Wechsler Abbreviated Scale of Intelligence (WASI) block design, Wechsler Memory Scale (WMS) Visual reproduction, and Delis Kaplan Executive Function System (DKEFS).

#### 2.3.3. Physical Measures

Physical abilities including, gait, balance, and upper extremity function were assessed using the Upper Extremity Fugl Myer (UEFM), Berg Balance test, and GaitRite computerized system. Gait velocity, step length, and step time were measured with the GaitRite system.

#### 2.3.4. Quality of Life and Stroke Recovery

Health status following a stroke was assessed for this study using the Stroke Impact Scale, which measures 8 different domains: strength, hand function, ADL/IADL, mobility, communication, emotion, memory/thinking, and participation [41, 42]. In addition to the Stroke Impact Scale, items from the NIH funded Patient-Reported Outcomes measurement Information System (PROMIS) were used to measure pain, fatigue, sleep, and satisfaction with participation in social roles and activities [43–45]. The Beck Depression Inventory-2 (BDI) was used to assess depression.

#### 2.3.5. Biomarkers

Potential biomarkers for treatment and recovery were assessed using ELISA's for astrogliosis (GFAP, Aplco Immunoassays, Salem, NH), astrocytic damage (S100*β*, Aplco Immunoassays, Salem, NH), neuronal damage (Neuron specific enolase, Aplco Immunoassays, Salem, NH), and neuroinflammation (IL-6, TNF-*α*, R&D Systems, Minneapolis, MN). These factors have been shown in animal and human studies to be regulated by neural injury and recovery and measurable in plasma or serum [46–48]. These potential biomarkers were chosen on the basis that previous studies have observed an effect of HBOT on neuroprotection and inflammation (for review see Background, or [6, 17, 20, 27, 28]).

### 2.4. Data Analysis

First, we established the stable baseline by comparing Baseline 1 and Baseline 2. Only the SIS global and sleep measures (part of Quality of Life) revealed reliable improvement across the two baseline assessments. Next, Cohen's d effect sizes were calculated for each measure by combining the two baseline data points and the two treatment data points: Cohen's d = (M_Treatment1,2_ - M_Baseline1,2_) / SD. In addition, the dependent means t test was reported for each effect size. All t tests were two-tailed and based on 5 degrees of freedom (df) except for gait velocity, which was based on 4 df. In domains where the effect size was greater than 0.5, which is typically considered a large effect, additional contrasts based on repeated measures ANOVA were used to test patterns in the data. Consistent with the t tests, we compared (Baseline 1 and Baseline 2) with (Treatment 1 and Treatment 2) to determine whether improvement is associated with the intervention. Next, to determine the longevity of treatment effects we compared (Treatment 1 and Treatment 2) with (Off and Follow-Up), which represent 1 month without treatment and 3 months without treatment, respectively. Finally, Off and Follow-Up were contrasted against each other to examine fading of gains over time. To assure that the effects observed are the result of the physiologic intervention and not another factor such as a practice effect, we calculated a linear trend score to reflect steady improvement over Treatment 1 (contrast coefficient, -3), Off (coefficient score, -1), Treatment 2 (contrast coefficient, +1), and Follow-Up (contrast coefficient, +3), as would be expected due to practice. Perfect maintenance of gains would result in a flat function; continuing improvement even in the absence of treatment would suggest a positive contrast score. We did not assess the linear trend over the entire study because that set of contrast coefficients correlated too highly with a pure treatment effect. Further, we did not include the biomarkers in this analysis because previous analyses had already demonstrated that practice could not account for those patterns. All analysis was completed with JMP software.

## 3. Results

To examine the effect of HBOT following an ischemic stroke we utilized a within design where each subject provided his or her own baseline. A total of 7 subjects were enrolled in this study. One patient withdrew due to ear pain associated with the HBOT, but this was the only adverse event noted during this study. One participant did not return for the 3-month follow-up visit. All subjects experienced an ischemic stroke in the right hemisphere of the brain (Table 1). Of the remaining 6 participants, 4 were female and 2 were male with ages ranging from 31 years to 79 years.

### 3.1. Language Domains

To investigate the potential effect of HBOT on aphasia, communication ability, language, and verbal fluency the RCBA and BNT were used. We did not identify significant deficits at baseline in these speech and language domains; therefore, as we would not expect differences when comparing treatment to baseline no further analysis was completed.

### 3.2. Neuropsychological Domains

An extensive neuropsychological battery was used to measure changes in cognitive impairments (e.g., memory, attention/concentration, and verbal fluency). Using the MMSE we did not observe impairments in general cognition for any participant (average score before treatment = 28.6 out of 30 possible points). Therefore, we did not complete further analysis on the MMSE. Overall, mild to moderate impairments were observed in the various cognitive domains measured. The baseline was stable for all the cognitive assessments. We observed a significant effect of treatment on verbal and nonverbal memory. Specifically, we observed a significant effect of HBOT on the CVLT, which measures verbal memory, and the WMS, which measures nonverbal memory (Figure 1, Tables 2 and 3). We confirmed the treatment effect as compared with the pretreatment baseline and were able to determine that this effect was maintained through the 3-month follow-up (Table 3). A linear trend score of 3.2 (t=0.13) was identified for the CVLT and -1.4 (t=-0.17) for the WMS suggesting no practice effect. Although the effect size of 0.8 suggests a potential effect on processing speed and executive function, as measured with Trails A and the DKEFS, we did not observe a significant treatment effect in these domains or on language or visuospatial abilities in these participants (Tables 2 and 3).

### 3.3. Physical Function

Gait, balance, and upper extremity movement were compared before and after HBOT treatment. We observed significant improvement in physical abilities as measured with the Upper Extremity Fugl Meyer (UEFM) and gait velocity. Gait velocity, as reported by the %-normalized to the general population increased nearly 20%, this effect was maintained at follow-up (Figure 2, Tables 2 and 3). Other aspects of gait were measured such as step length and cycle time but we only observed significant changes when examining gait velocity. We also measured balance using the Berg Balance scale but did not observe any changes at any of the testing points throughout the study. The linear trend scores for all the physical domains were all near zero again suggesting little to no practice effect (t = -0.133 - .33).

### 3.4. Quality of Life

Using the PROMIS QOL measure, participants reported significant improvement in sleep following HBOT and at the 3-month follow-up (Figure 3, Tables 2 and 3). Significant improvement in overall recovery as measured with the SIS was also reported following HBOT and at 3 months following treatment (Figure 3, Tables 2 and 3). A linear trend score of -11 (t= -1.1) was identified for sleep while a mean linear score of 41 (t=1.8) was noted for global recovery. In all but 1 patient the linear contrast for global recovery was positive suggesting this effect due to more than the treatment. While we observed a decrease in depression levels this effect did not reach significance. No other significant differences were noted as a result of treatment on individual domains related to QOL or recovery using the PROMIS or SIS, such as hand function, satisfaction, or activities of daily living.

### 3.5. Biomarkers

To examine physiological biomarkers for treatment and recovery neural, glial, and inflammatory markers were measured. The strengths of these relationships are displayed with the effect size, which range from .69 to 1.5. A significant treatment effect was observed for 3 of the physiological biomarkers measured, NSE, TNF-alpha, and IL-6 (Tables 2 and 3). While these biomarker values appeared to be returning to baseline levels at both the 1 month off and 3-month follow-up, a significant effect was only observed for NSE and TNF-a (Table 3).

## 4. Discussion

The purpose of this study was to investigate the role of HBOT as a therapeutic intervention for stroke patients. A stroke may result in a variety of functional deficits including physical, cognitive, and behavioral impairments. Using a within-subject design, we measured the impact of HBOT across a number of functional domains including speech, language, cognition, physical function, emotional / behavior impairments, and quality of life. In this preliminary study, our approach was to identify effects that were strong enough to emerge with a very small sample and, then, to examine the nature of those effects. For example, whether or how quickly they fade and whether they can be attributed to practice. This approach is likely to underestimate the potential for hyperbaric treatment due to the low statistical power for identifying effects strong enough for examination. However, the consistency of improvement noted over repeated assessments spread out over months argues against any account based on statistical fluke. Significant improvements following HBOT were observed with cognition (including, memory, and processing speed), gait velocity, upper extremity mobility, sleep, and overall recovery, as measured with the SIS. These treatment effects were maintained when examined at 3 months following treatment with the potential exception of the UEFM. We also observed a significant change in neural and inflammatory biomarker expression levels in response to HBOT. The pattern observed for the biomarkers was different than all the functional measures suggesting transient physiological responses but sustained functional change.

Although we observed significant improvements in cognition and gait velocity, there are limitations to interpretation. For example, while there was an increase in gait velocity from baseline to treatment other gait kinematics such as stride length and step length which compare symmetry of the left to right side did not show significant differences; the participants were just walking at a faster speed maintaining their biomechanical deficits. Furthermore, there were no significant differences on the Berg Balance test, which may indicate the increases in gait velocity may not be due to improvements of motor control of the paretic limb. In general the testers suggested that participants might have been trying to perform better at each testing interval, as they were not blinded to treatment. However, there is evidence suggesting a minimal practice effect with the CVLT and WMS [49, 50]. Further, a linear trend score was calculated for each significant effect to differentiate between a treatment effect and other confounding variables such as a practice effect. Continuing improvement even in the absence of treatment was signified by a positive contrast score, which we only observed for global recovery. Therefore, the statistical improvement in quality of life demonstrated by the SIS may have been in part due to the treatment but may also have been an effect of increased attention paid by clinical staff or another indirect result of participation in this study. Social isolation is very common with a long-term disability along with significant loss of self-worth, income, independence, and many other domains, thus a sustained increase in global improvement, participation, and emotional wellbeing are of particular note despite the cause. Changes in the physiological biomarkers suggest measureable differences are occurring following HBOT however in this current study we are unable to clearly define the link between these biomarkers and functional changes. However, one remarkable feature of these results is that the biomarker measures are so sensitive to the presence and absence of hyperbaric treatment, even for patients in the chronic stages of their illness.

The use of HBO as a treatment following stroke was first raised 40–50 years ago [32, 51]. Despite decades of interest in HBOT previous studies investigating the effects of HBOT following a stroke have produced mixed results [10, 11, 18, 19, 32, 40, 52–60]. There are a number of variables that may account for the incongruous literature including several related to the study design including the treatment protocol, study population, inclusion of and type of control group, outcomes measured, and timing of treatment. An update from a 2014 Cochrane review reported that when taken together the existing literature does not find HBOT an effective intervention in the acute phase following an ischemic stroke [2, 10]. However, statistically significant improvement was noted in functional outcomes in some of the studies [10]. Our findings fall in line with the literature as we did observe significant effects on some domains but there were also a number of areas where no functional change was observed. Consistent with our observation that HBOT improves memory is a recent retrospective study that also observed significant improvement in verbal and nonverbal memory [39]. Despite differences in the time from stroke onset and treatment protocol as well as differences in the measures for verbal and nonverbal memory, the percent change, around 20%, was similar between both studies. Using single-photon emission computed tomography (SPECT) they posit that their memory changes correlate with metabolic changes in the brain [39]. A recent randomized controlled trial with patients 1 year after stroke also used SPECT to begin to elucidate potential mechanisms of action of HBOT [40]. Specifically, the authors reported changes in brain metabolism observed with SPECT that correlated with quality of life, the NIHSS, and activities of daily living [40]. Comparably, we observed changes in overall global recovery and some aspects related to quality of life, such as sleep in our similar study population with participants that were on average 1.5 years after stroke. We also endeavored to look at mechanism by looking at blood biomarkers for neural activity (GFAP, NSE) and inflammation (TNF-a, IL-6). We found transient changes in the expression levels of these markers suggesting the potential role of the oxygen in modulating neural and inflammatory signaling cascades, which may lead to the sustained functional changes we observed. Animal models have outlined multiple possibilities for the role of HBOT on antiapoptotic and anti-inflammatory signaling pathways including Nogo-A, bcl-2 for plasticity and TNF, IL-1, IL-6, and COX-2 for inflammation [15, 17, 30, 61–73]. However, with blood based markers that are found throughout the body we are unable to make definitive statements about mechanism at this time.

Adding to the studies investigating the effects of HBOT following stroke, with their mixed results, there has been a strong recent interest in the effectiveness of HBOT following a TBI, due to the increase in brain injuries sustained during recent military combat conflicts. The Department of Defense is implementing many facilities whose purpose is to use hyperbaric oxygen therapy to help veterans recover from TBI. A number of case studies have supported the use of HBOT following a TBI, suggesting beneficial effects even years after injury [12]. In a large single center double blind randomized sham controlled prospective trial at the US Air Force School of Aerospace medicine the effects of 2.4 atmospheres of HBO was assessed in 50 military service members [74]. The control group received 1.3 ATA HBO. While some measures were improved following HBOT, they were improved in both the 2.4 and 1.3 ATA groups, which has resulted in discussion regarding the appropriateness of 1.3 ATA as a control or rather should it be seen as a low dose of HBOT [12]. Interestingly, in a study of 60 service members at the Naval medicine operational training center in Naval Air station Pensacola no differences were found when comparing oxygen at 1.5 and 2.0 ATA for individuals with mild TBI [75]. In our study we used 2.0 ATA and found similar results on cognition and aspects of recovery and QOL after a stroke as these studies with did with varying doses of HBOT following TBI. For example, 1.5 ATA of oxygen was used for individuals with mild TBI and significant changes were noted in cognition and QOL [76]. However this study, similar to ours, did not have a control group that was blinded to condition. The limitations regarding different types of controls groups continue to make it challenging to clearly ascertain the role of HBOT following neurologic injury.

Other confounding variables including the type and sensitivity of outcome measures or domains assessed, and the timing of HBOT may all play a role in the incongruous and inconsistent findings in the literature. Due to the nature of ischemic injury one may conclude that HBOT would be most effective during acute injury when neurons can be rescued in addition to modulating plasticity and synaptic changes in new or existing neurons to compensate. Due to normal recovery during the initial 6–12 months following injury it is difficult to assign responsibility to one intervention. Despite the preponderance of evidence for an acute timeframe, Boussi et al. suggest neuroplasticity is possible in patients as far as 5 years after traumatic brain injury [76]. Based on the changes we observe in biomarker expression levels, we also support the idea that neural changes are occurring years following an injury, as well as inflammatory changes, which may lead to downstream signaling cascades [76]. Still, we are unable to definitely link changes in physiological biomarkers to functional changes, which may be more evident if HBOT is used acutely similar to pharmacologic interventions that complement to the rehabilitative process.

We appreciate that there are several limitations to the design of this study, some of which have been discussed above. While the within-subject design eliminates some of these issues previously discussed with RCT, the small sample size and lack of blinding and controls limit the generalizability of the results. Prior research has demonstrated the significant subject and observer/researcher bias inherent in this type of research, specifically with HBOT, and thus interpretation of the results its overall contribution to the scientific are narrow. Potentially the greatest limitation is only including subjects who have experienced a plateau in their recovery. This time frame places our study at risk of missing a critical therapeutic window to rescue cells before they are no longer viable, suggesting that the effects we hope to observe from HBOT treatment would be due to other mechanisms, i.e., not classical neuroprotection pathways. However, this time frame is necessary for this study design as the baseline must be stable to compare treatment effects. This is where other observational studies and even controlled clinical trials have fallen short and why the results from those studies are ultimately ineffectual. However, despite the limitations that can be found in most experimental design, the growing body of literature provides new and reliable data helping us to better understand the effects of HBOT on impairments resulting from ischemic strokes.

## 5. Summary

This study investigated the impact of HBOT as a therapeutic intervention following stroke across a number of functional domains including speech, language, cognition, physical function, and quality of life. We found a beneficial effect of HBOT on memory, processing speed, gait velocity, upper extremity mobility, sleep, and overall recovery. We also observed significant transient changes in neural and inflammatory biomarkers in response to HBOT that may result in the sustained functional changes that were observed. Despite these encouraging results further research is needed to more clearly define the mechanism and potential role of HBOT following stroke.

## Figures and Tables

**Table 1:** Subject demographics.

***Subject ID***	***Gender***	***Age***	***Time from Injury***	***Type of Stroke***
**1**	Male	79	2 years	Ischemic R MCA
**2**	Male	61	1 year	Ischemic R MCA
**3**	Female	57	1 year	Ischemic R MCA
**5**	Female	57	2 years	Ischemic R MCA
**6**	Female	59	2 years	Ischemic R MCA
**7**	Female	31	1 year	Ischemic R MCA

**Table 2:** Effect of HBOT on functional impairments: means and effect size.

***Domain***	***Baseline*** *(mean+ SD)*	***HBOT*** *(mean+ SD)*	***Paired t test*** *B1,2 vs T1,2*	***Effect Size***
**Cognitive**				
CVLT-II	49.6 + 10.4	59 + 11.9	0.01*∗*	1.56
GP	39.4 + 9.5	44.5 + 15.5	0.13	0.38
Trails A	34.1 + 8.6	39.2 + 7.4	0.10	0.79
Trails B	37.9 + 12.6	40.8 + 8.4	0.51	0.28
COWAT	38.1 + 8.3	41.7 + 9.6	0.09	0.81
SF - animals	42.9 + 10.8	44.1 + 15.2	0.76	0.13
WASI	44.1 + 12.5	44.6 + 9.5	0.78	0.11
WMS	10.4 + 0.9	12.2 + 2.1	0.03*∗*	1.11
DKEFS	9.7 + 2.9	11.1 + 1.6	0.11	0.76

**Physical**				
UEFM	37.7 + 23.1	42.0 + 22.2	0.03*∗*	0.85
Berg	48.7 + 9.7	50.7 + 9.8	0.26	0.5
Gait Velocity	68.7 + 8.7	83.5 + 8.4	0.01*∗*	1.5
Step length	3.79 + 1.5	2.36 + 1.9	0.33	-0.39
Step time	0.05 + 0.06	0.04+ 0.02	0.36	-0.36

**Quality of Life **				
BDI	14.5 + 10.8	10.0 + 7.7	0.09	-0.82
SIS Global	52.1 + 26.5	61.7 +18.4	0.04*∗*	0.73
SIS Strength	49.0 + 13.6	49.5 + 13.5	0.75	0.04
SIS Memory	85.9+ 17.7	90.9 + 12.8	0.59	0.34
SIS Emotional	74.4 + 15.8	82.6 + 13.8	0.14	0.54
Communication	84.8 + 20.1	86.8 + 12.4	0.65	0.28
SIS ADL's	72.3 + 19.3	72.5 + 19.9	0.95	0.02
SIS mobility	71.2 + 10.9	78.3 + 15.8	0.32	0.38
Hand Function	58.9 + 32.5	60.6 + 31.8	0.57	0.23
Participation	50.6 + 13.8	64.6 + 30.1	0.11	0.54
Physical Comp.	56.0 + 25.8	61.1 + 24.4	0.09	0.81
Sleep	41.2 + 7.7	48.5 + 9.8	0.04*∗*	1.17
Satisfaction	46.2 + 6.1	53.8 + 12.2	0.16	0.68

**Biomarkers**				
NSE	2.2 + 0.4	2.9 + 0.5	0.005*∗*	2.1
GFAP	10.1 + 18.9	17.3 + 27.5	0.13	0.74
IL-6	6.3 + 6.8	3.9 + 4.9	0.05*∗*	-1.0
TNF-a	7.0 + 2.0	4.7 + 1.4	0.01*∗*	-1.5

**Table 3:** Effect of HBOT on functional impairments: repeated measures ANOVA.

***Domain***	***B1 versus B2***	***B1,2 vs T1,2***	***T1,2 vs Off and Follow-up***	***Off vs Follow-up***
**Cognitive**				
CVLT-II	F_(1,5)_=0.13, p=0.74	F_(1,5)_=15.7, p=0.01	F_(1,4)_=0.13, p=0.74	F_(1,4)_=2.78, p=0.19
Trails A	F_(1,5)_=0.01, p=0.77	F_(1,5)_=3.9, p=0.10	F_(1,4)_=1.27, p=0.34	F_(1,4)_=2.95, p=0.18
COWAT	F_(1,5)_=2.59, p=0.17	F_(1,5)_=4.0, p=0.09	F_(1,4)_=0.31, p=0.61	F_(1,4)_=0.003, p=0.96
WMS	F_(1,5)_=0.29, p=0.61	F_(1,5)_=7.74, p=0.03	F_(1,4)_=1.0, p=0.39	F_(1,4)_=1.0, p=0.39
DKEFS	F_(1,5)_=0.11, p=0.78	F_(1,5)_=3.6, p=0.11	F_(1,4)_=0.07, p=0.81	F_(1,4)_=0.11, p=0.76

**Physical**				
UEFM	F_(1,5)_=0.65, p=0.46	F_(1,5)_=9.6, p=0.03	F_(1,4)_=6.5, p=0.06	F_(1,4)_=0.24, p=0.64
Gait Velocity	F_(1,4)_=3.7, p=0.12	F_(1,4)_=24.9, p=0.01	F_(1,3)_=0.13, p=0.75	F_(1,3)_=0.25, p=0.65

**Quality of Life **				
BDI	F_(1,5)_=0.16, p=0.71	F_(1,5)_=6.7, p=0.09	F_(1,4)_=1.4, p=0.32	F_(1,4)_=0.66, p=0.48
SIS Global	F_(1,5)_=8.4, p=0.03	F_(1,5)_=7.2, p=0.04	F_(1,4)_=2.0, p=0.23	F_(1,4)_=1.67, p=0.27
SIS Emotional	F_(1,5)_=0.56, p=0.28	F_(1,5)_=3.0, p=0.14	F_(1,4)_=0.83, p=0.41	F_(1,4)_=0.51, p=0.51
SIS Participation	F_(1,5)_=0.005, p=0.9	F_(1,5)_=3.7, p=0.11	F_(1,4)_=0.56, p=0.50	F_(1,4)_=0.08, p=0.80
SIS Physical	F_(1,5)_=2.8, p=0.17	F_(1,5)_=4.2, p=0.09	F_(1,4)_=0.96, p=0.38	F_(1,4)_=1.0, p=0.36
Sleep	F_(1,5)_=6.5, p=0.06	F_(1,5)_=8.2, p=0.04	F_(1,4)_=1.5, p=0.28	F_(1,4)_=0.001, p=0.97
Satisfaction	F_(1,5)_=0.06, p=0.82	F_(1,5)_=2.7, p=0.16	F_(1,4)_=6.8, p=0.06	F_(1,4)_=0.11, p=0.75

**Biomarkers**				
NSE	F_(1,5)_=0.01, p=0.85	F_(1,5)_=22.8, p=0.005	F_(1,4)_=16.0, p=0.01	F_(1,4)_=0.24, p=0.64
GFAP	F_(1,5)_=0.06, p=0.81	F_(1,5)_=3.27, p=0.13	F_(1,4)_=2.97, p=0.16	F_(1,4)_=0.13, p=0.74
IL-6	F_(1,5)_=0.02, p=0.89	F_(1,5)_=6.0, p=0.05	F_(1,4)_=5.3, p=0.08	F_(1,4)_=1.1, p=0.36
TNF-a	F_(1,5)_=0.04, p=0.85	F_(1,5)_=14.2, p=0.013	F_(1,4)_=18.4, p=0.01	F_(1,4)_=0.34, p=0.59

## Data Availability

The data from this study is not an archived dataset. It was a small case series study.
